# Efficacy and prognostic predictors of primary systemic therapy for de novo Stage IV breast cancer. Exploratory analysis of JCOG1017 PRIM-BC

**DOI:** 10.1007/s12282-025-01821-4

**Published:** 2026-01-31

**Authors:** Tadahiko Shien, Makoto Ishitobi, Keita Sasaki, Kiyo Tanaka, Akihiko Shimomura, Takahiro Tsukioki, Yasuaki Sagara, Yuri Kimura, Kaori Terata, Takayuki Ishiba, Hiroyuki Yasojima, Makiko Ono, Kyoko Goda, Yoichi Naito, Tomomi Fujisawa, Fumikata Hara, Gakuto Ogawa, Haruhiko Fukuda, Hiroji Iwata

**Affiliations:** 1 Department of Breast and Endocrine Surgery, Okayama University Hospital, 2-5-1 Shikata-Cho, Kita-Ku, Okayama, 7008558 Japan; 2 Breast Surgery, Osaka Habikino Medical Center, Osaka, Japan; 3 JCOG Operation Office/Data Center, National Cancer Center Hospital, Tokyo, Japan; 4 Breast Surgery, Toranomon Hospital, Tokyo, Japan; 5 Breast and Medical Oncology Department, NCGM - Center Hospital of the National Center for Global Health and Medicine, Tokyo, Japan; 6 Breast Surgical Oncology Department, Sagara Hospital, Kagoshima, Japan; 7 Breast Surgery, Japanese Foundation for Multidisciplinary Treatment of Cancer, Tokyo, Japan; 8 Breast and Endocrine Surgery, Akita University Hospital, Akita, Japan; 9 Breast Surgery, University of Science Tokyo, Tokyo, Japan; 10 Breast Surgery, Osaka Medical Center, Osaka, Japan; 11 Department of Medical Oncology, The Cancer Institute Hospital of JFCR, Tokyo, Japan; 12 Breast Surgery, Kanagawa Cancer Center, Yokohama, Japan; 13 Department of Developmental Therapeutics, National Cancer Center Hospital East, Kashiwa, Japan; 14 Breast Oncology, Gunma Prefectural Cancer Center, Gunma, Japan; 15 Breast Oncology, Aichi Cancer Center Hospital, Nagoya, Japan; 16 Department of Advanced Clinical Research and Development, Nagoya City University Graduate School of Medical Sciences, Nagoya, Japan

**Keywords:** Metastatic Breast Cancer, de novo Stage IV, Primary systemic therapy

## Abstract

The optimal initial systemic therapy for de novo Stage IV breast cancer (BC) is critical, particularly to assess early drug efficacy. In the JCOG1017 trial, untreated de novo Stage IV BC patients received 3 months of subtype-based systemic therapy before randomization for primary tumor resection. Among 569 treated patients, the overall non-progression disease (non-PD: no 10% or more increase in maximum diameter) rate was 77.2%, and the objective response rate (ORR) was 29.0%. By subtype, non-PD/ORR rates were TN (78.2%/36.4%), Luminal (75.4%/13.4%), HER2 (92.9%/81.0%), and Luminal-HER2 (66.7%/40.3%). Multivariable analysis revealed higher non-PD rates in postmenopausal patients (odds ratio (OR) 1.673, p = 0.024) and those with PgR-positive tumors (OR 2.391, p = 0.0019). Endocrine therapy was more effective for patients with PgR-positive tumors (OR 3.258, p < 0.0001) and less practical in those with visceral metastasis (OR 0.605, p = 0.0334). HPD (Trastuzumab, Pertuzumab, Docetaxel)/HPTX (Trastuzumab, Paclitaxel) therapies (non-PD rate: 92.7%) were highly effective for visceral metastasis (OR = 15.818, p = 0.0030). Patients without progression at 3 months had significantly better overall survival (HR 0.508, 95% CI [0.398, 0.648]). These findings emphasize that initial therapy must align with subtype, and endocrine monotherapy is suboptimal for ER + HER2 + BC. Effective strategies are needed for those with early progression.

**Supplementary Information:**

The online version contains supplementary material available at 10.1007/s12282-025-01821-4.

## Introduction

There are various guideline recommendations for the appropriate drug treatment of metastatic recurrent breast cancer (BC). The treatment of metastatic BC is based on subtypes classified by ER (estrogen receptor), PgR (progesterone receptor), and HER2 (human epidermal receptor-2) expression. Untreated de novo Stage IV patients are more likely to respond to systemic therapy than patients diagnosed with postoperative recurrent BC [1]. Many clinical trial data reported to date for the first-line treatment of metastatic BC are mainly for patients with recurrent BC. It is unclear whether tumor response differs depending on each BC subtype or tumor location, such as primary site or distant metastasis, and whether the tumor response to primary systemic therapy (PST) affects the prognosis in patients with de novo stage IV BC. There is also limited data on predictive factors of tumor response to each treatment and prognostic factors for de novo stage IV BC.

In JCOG1017, a randomized trial comparing primary tumor resection (PTR) plus systemic therapy with systemic therapy alone in de novo Stage IV BC patients, de novo Stage IV patients were treated with PST for 3 months according to BC subtypes before randomization [2] We already presented the results of the primary analysis [3]. PTR could not significantly prolong the overall survival of de novo Stage IV BC patients with sensitivity to PST (median overall survival 75 months vs. 69 months, HR 0.86, 90% CI 0·69–1·07, one-sided p = 0·13). In this trial, we uniformly evaluated sensitivity to PST at 3 months for all subtypes and evaluated the survival. Therefore, we aimed to obtain data on the efficacy of PST and the prognosis of only de novo Stage IV BC.

In this exploratory analysis, we examined the efficacy and predictive factors of response to each drug in de novo Stage IV BC and whether assessing sensitivity to all drugs is appropriate. We also want to clarify the efficacy of PST for de novo Stage IV patients and develop optimal treatment strategies for de novo Stage IV BC using data from JCOG1017.

## Patients and methods

### Study design and participants

**Fig. 1:**
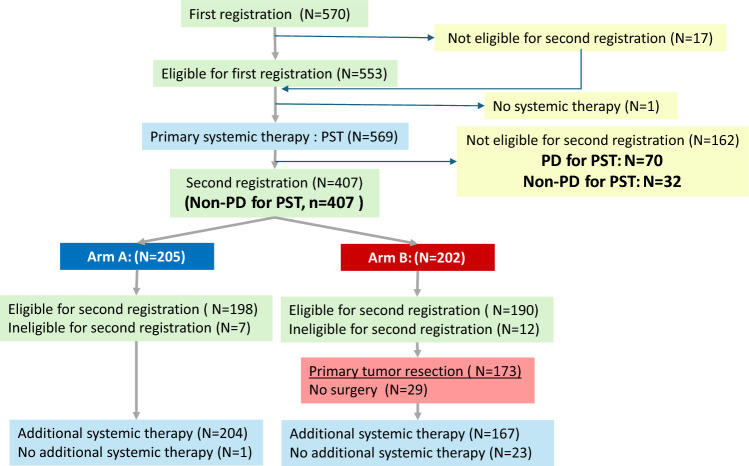
CONSORT diagram

JCOG1017 is a multicenter, open-label, randomized, controlled phase 3 trial at 44 institutions in Japan (Appendix 1) designed to confirm the superiority of PTR in addition to systemic therapy for overall survival over systemic treatment alone. This trial was conducted according to the Declaration of Helsinki, the Japanese Ethical Guidelines for Clinical Research, and the Clinical Trials Act. The Japan Clinical Oncology Group (JCOG) Protocol Review Committee and the National Cancer Center Hospital East Certified Review Board approved the study protocol. All patients provided written informed consent before enrolment. Full trial details have been published previously; the CONSORT diagram is shown in Fig. 1 [2].

The eligible patients were aged 20–80 years with an Eastern Cooperative Oncology Group (ECOG) performance status of 0 or 1 (PS 2 caused by the symptoms of bone metastasis is also eligible), histologically proven invasive BC, and without bilateral BC or extension to the contralateral breast. The patients with at least one measurable metastatic lesion other than the breast tumor and axillary lymph nodes, and without brain metastasis, were included. The second registration and randomization were performed for patients without progression after PST. This exploratory analysis was conducted on patients enrolled in the first registration and who underwent PST.

### Procedures

All enrolled patients for the first registration received PST. The PST regimen was decided according to the ER and HER2 status and the disease situation (Appendix 2). ER-positive (> 10%) patients, regardless of HER2 status, with no life-threatening diseases received endocrine therapy, which was oral tamoxifen 20 mg/body daily plus subcutaneous goserelin 3·6 mg/body (day 1) every 4 weeks for premenopausal patients and oral letrozole 2·5 mg/body daily for 4 weeks for post-menopausal patients. CDK4/6 inhibitors were not used because they were preapproved during this trial. Patients with ER-negative and/or life-threatening diseases received the following chemotherapy; for HER2-positive (IHC 3 + or ISH +) patients, intravenous paclitaxel 80 mg/m^2^ (days 1, 8, 15) plus weekly trastuzumab 2 mg/kg (days 1, 8, 15, 22) every 4 weeks (before Feb. 3. 2014) (HPTX)or intravenous docetaxel 75 mg/m^2^ (day1) plus trastuzumab 6 mg/kg (day1), pertuzumab 420 mg/body (day1) every 3 weeks (after Feb. 3. 2014 when reported the results of CLEOPATRA study [4]) (HPD), and for HER2-negative patients’ intravenous paclitaxel 80 mg/m^2^ (days 1, 8, 15) every 4 weeks. Pembrolizumab was not used to treat triple-negative BC because it was not approved at the time of this trial. After each regimen, tumor assessment was conducted after 3 months, while the docetaxel, trastuzumab, and pertuzumab regimens underwent evaluation after four cycles. In the PTR plus systemic therapy arm, the patients underwent the complete resection of the primary breast tumor within 42 days after the second registration. Adjuvant radiotherapy after partial or total mastectomy was not allowed. After surgery, the same systemic therapy was continued as soon as possible. In the systemic therapy alone arm, the patients continued the same systemic therapy after the second registration.

### Outcomes

The non-PD (Progressive Disease) rate and the response rate were examined.

In the non-PD rate, PD was defined as meeting any of the following criteria after 3 months of systemic therapy: (i) the diameter of the primary tumor lesion increased 10% or more compared to the most minor recorded diameter, (ii) the sum of the diameters of distant metastases increased 10% or more compared to the smallest recorded sum of the diameters, (iii) unequivocal progression of existing non-target lesions, (iv) appearance of new malignant lesions.

The response rate was defined as a proportion of patients with complete response (CR) or partial response (PR) according to Response Evaluation Criteria in Solid Tumors (RECIST) ver. 1.1 [5]. We considered patients with CR or PR as responders, and patients with stable disease or progression disease, defined by RECIST ver 1.1, non-responders.

The overall survival of responders vs. non-responders and the PD vs. non-PD group was also evaluated. Overall survival was defined as the number of days from the date of randomization to the date of death from any cause.

### Statistical analysis

The non-PD and response rates were reported as percentages and 95% confidence intervals (CIs) calculated by the Clopper-Pearson method. To examine the predictive factors for the non-PD rate and the response rate for all the first registered patients and each drug, univariable and multivariable logistic regression models were performed with the following clinical factors as covariates: menopausal status (pre/post), cT (cT1/ cT3/ cT3/ cT4), cN (cN0/ cN1/ cN2/ cN3), visceral mets (yes/no), histology (IDC/special types), ER (negative/positive; univariable only), PgR (negative/positive; univariable only), HER2 (negative/positive) and subtype (ER (−) and HER2 (−)/ ER ( +) and HER2 (−/ ER (−) and HER2 ( +)/ ER ( +) and HER2 ( +)). OS was estimated with the Kaplan–Meier method. The univariable analyses were performed with the Cox proportional hazard model to calculate the hazard ratio between the responders and non-responders, as well as PD and non-PD. All statistical analyses were conducted using SAS software (version 9.4).

## Results

Of 570 de novo Stage IV patients enrolled in the JCOG1017, 569 patients who received PST were examined in this exploratory analysis. 372 patients with ER + /non-life-threatening metastases received endocrine therapy (tamoxifen + LH-RH agonist or letrozole), 109 patients with ER-/HER2 + or ER + /HER2 + / life-threatening metastases received HPD or HPTX and 88 patients with ER-/HER2- tumors received PTX. The details of the clinical and pathological features are in Table 1. Median age was 57, and 198 (34.8%) patients were premenopausal.

**Table 1:** Patient characteristics

	Tam + LH-RH or LET	HPD or HPTX	PTX	Total
	*n* = 372	*n* = 109	*n* = 88	*n* = 569
Age Median (min–max)	58 (23–79)	55 (31–79)	57 (25–80)	57 (23–80)
*Menopausal status*				
Premenopausal	130 (34.9)	35 (32.1)	33 (37.5)	198 (34.8)
Postmenopausal	242 (65.1)	74 (67.9)	55 (62.5)	371 (65.2)
*cT*				
cT1	26 (7.0)	4 (3.7)	5 (5.7)	35 (6.2)
cT2	165 (44.4)	50 (45.9)	36 (40.9)	251 (44.1)
cT3	42 (11.3)	19 (17.4)	8 (9.1)	69 (12.1)
cT4	139 (37.4)	36 (33.0)	39 (44.3)	214 (37.6)
*cN*				
cN0	47 (12.6)	7 (6.4)	8 (9.1)	62 (10.9)
cN1	169 (45.4)	47 (43.1)	27 (30.7)	243 (42.7)
cN2	55 (14.8)	15 (13.8)	16 (18.2)	86 (15.1)
cN3	101 (27.2)	40 (36.7)	37 (42.0)	178 (31.3)
*Visceral metastasis*				
No	185 (49.7)	31 (28.4)	27 (30.7)	243 (42.7)
Yes	187 (50.3)	78 (71.6)	61 (69.3)	326 (57.3)
*Histology*				
Invasive ductal carcinoma	334 (89.8)	104 (95.4)	80 (90.9)	518 (91.0)
Special types	38 (10.2)	5 (4.6)	8 (9.1)	51 (9.0)
*ER*				
Negative	0 (0.0)	84 (77.1)	55 (62.5)	139 (24.4)
Positive	372 (100.0)	25 (22.9)	33 (37.5)	430 (75.6)
*PgR*				
Negative	68 (18.3)	93 (85.3)	59 (67.0)	220 (38.7)
Positive	304 (81.7)	16 (14.7)	29 (33.0)	349 (61.3)
*HER2*				
Negative	325 (87.4)	0 (0.0)	88 (100.0)	413 (72.6)
Positive	47 (12.6)	109 (100.0)	0 (0.0)	156 (27.4)
*Subtypes*				
ER (−) HER2 (−)	0 (0.0)	0 (0.0)	55 (62.5)	55 (9.7)
ER ( +) HER2 (−)	325 (87.4)	0 (0.0)	33 (37.5)	358 (62.9)
ER (−) HER2 ( +)	0 (0.0)	84 (77.1)	0 (0.0)	84 (14.8)
ER ( +) HER2 ( +)	47 (12.6)	25 (22.9)	0 (0.0)	72 (12.7)

Table 2 shows the tumor response of PST for 3 months. The non-PD rate for all patients was 77.2% (439/569, 95%CI 73.5–80.5%), and the predictive factors of non-PD were post-menopausal (odds ratio [OR] 1.673, 95%CI 1.070–2.617, p = 0.024), PgR positive (OR 2.391, 95%CI 1.380–4.140, p = 0.0019), and subtypes in multivariable analysis (Table 2.1).

**Table 2:** Univariable and Multivariable analysis of predictors to PST

Factor		Non-PD/N	Non-PD Rate (95% CI)	Univariable analysis		Multivariable analysis	
				Odds ratio (95% CI)	*P*-value	Odds ratio (95% CI)	*P*-value
*All patients*							
Menopausal status	Premenopausal	146/198	73.7% (67.0%–79.7%)	1		1	
	Postmenopausal	293/371	79.0% (74.5%–83.0%)	1.340 (0.896–2.004)	0.1544	1.673 (1.070–2.617)	0.0240
cT	cT1	25/35	71.4% (53.7%–85.4%)	1		1	
	cT2	199/251	79.3% (73.7%–84.1%)	1.565 (0.710–3.448)	0.2666	1.429 (0.630–3.239)	0.3932
	cT3	53/69	76.8% (65.1%–86.1%)	1.335 (0.534–3.339)	0.5366	1.502 (0.577–3.909)	0.4043
	cT4	162/214	75.7% (69.4%–81.3%)	1.275 (0.577–2.816)	0.5487	1.182 (0.511–2.732)	0.6956
cN	cN0	53/62	85.5% (74.2%–93.1%)	1		1	
	cN1	190/243	78.2% (72.5%–83.2%)	0.633 (0.296–1.351)	0.2369	0.562 (0.258–1.226)	0.1476
	cN2	62/86	72.1% (61.4%–81.2%)	0.453 (0.196–1.049)	0.0646	0.452 (0.188–1.083)	0.0748
	cN3	134/178	75.3% (68.3%–81.4%)	0.537 (0.248–1.164)	0.1154	0.454 (0.204–1.010)	0.0529
Visceral metastasis	No	188/243	77.4% (71.6%–82.5%)	1		1	
	Yes	251/326	77.0% (72.0%–81.5%)	0.981 (0.660–1.456)	0.9231	0.926 (0.608–1.411)	0.7210
Histology	Invasive ductal carcinoma	402/518	77.6% (73.8%–81.1%)	1		1	
	Special types	37/51	72.5% (58.3%–84.1%)	0.749 (0.393–1.429)	0.3801	0.768 (0.391–1.511)	0.4452
ER*	Negative	121/139	87.1% (80.3%–92.1%)	1			
	Positive	318/430	74.0% (69.5%–78.0%)	0.431 (0.252–0.737)	0.0021		
PgR	Negative	168/220	76.4% (70.2%–81.8%)	1		1	
	Positive	271/349	77.7% (72.9%–81.9%)	1.078 (0.723–1.607)	0.7139	2.391 (1.380–4.140)	0.0019
HER2*	Negative	313/413	75.8% (71.4%–79.8%)	1			
	Positive	126/156	80.8% (73.7%–86.6%)	1.330 (0.843–2.098)	0.2207		
Subtype	ER (−) & HER2 (−)	43/55	78.2% (65.0%–88.2%)	1		1	
	ER ( +) & HER2 (-)	270/358	75.4% (70.6%–79.8%)	0.878 (0.446–1.731)	0.7078	0.434 (0.193–0.978)	0.0439
	ER (−) & HER2 ( +)	78/84	92.9% (85.1%–97.3%)	3.468 (1.245–9.663)	0.0174	3.374 (1.210–9.407)	0.0201
	ER ( +) & HER2 ( +)	48/72	66.7% (54.6%–77.3%)	0.569 (0.255–1.268)	0.1676	0.345 (0.146–0.816)	0.0153
Overall		439/569	77.2% (73.5%–80.5%)				

Non-PD rate of endocrine therapy was 72.9% (271/372, 95%CI 68.0–77.3%), and that of tamoxifen + LH-RH agonist and letrozole were 68.5% (89/130, 95% CI 59.7–76.3%) and 75.2% (182/242, 95% CI 69.3–80.5%), respectively. The predictive factors of non-PD of endocrine therapy were visceral metastasis status (positive: OR 0.605, 95%CI 0.381–0.961, p = 0.033; reference group: negative), PgR status (positive: OR 3.258, 95%CI 1.886–5.628, p < 0.0001; reference group: negative) and HER2 status (positive: OR 0.365, 95%CI 0.195–0.684, p = 0.0016; reference group: negative). (Table 2.2).

**Table 2.2:** Patients received Endocrine therapy

Factor		Non-PD/N	Non-PD Rate (95% CI)	Univariable analysis	
				Odds ratio (95% CI)	*P*-value
cT	cT1	18/26	69.2% (48.2%–85.7%)	1	
	cT2	124/165	75.2% (67.8%–81.5%)	1.378 (0.561–3.389)	0.4844
	cT3	29/42	69.0% (52.9%–82.4%)	1.004 (0.350–2.879)	0.9940
	cT4	100/139	71.9% (63.7–79.2%)	1.169 (0.472–2.894)	0.7356
cN	cN0	39/47	83.0% (69.2%–92.4%)	1	
	cN1	124/169	73.4% (66.0%–79.9%)	0.589 (0.259–1.340)	0.2070
	cN2	38/55	69.1% (55.2%–80.9%)	0.473 (0.185–1.214)	0.1195
	cN3	70/101	69.3% (59.3%–78.1%)	0.482 (0.204–1.138)	0.0958
Visceral metastasis	No	144/185	77.8% (71.2%–83.6%)	1	
	Yes	127/187	67.9% (60.7%–74.5%)	0.605 (0.381–0.961)	0.0334
Histology	Invasive ductal carcinoma	244/334	73.1% (68.0%–77.7%)	1	
	Special types	27/38	71.1% (54.1%–84.6%)	0.885 (0.423–1.852)	0.7461
PgR	Negative	35/68	51.5% (39.0%–63.8%)	1	
	Positive	236/304	77.6% (72.5%–82.2%)	3.258 (1.886–5.628)	< 0.0001
HER2	Negative	246/325	75.7% (70.7%–80.3%)	1	
	Positive	25/47	53.2% (38.1%–67.9%)	0.365 (0.195–0.684)	0.0016
Overall		271/372	72.9% (68.0%–77.3%)		

The non-PD rate of PTX was 76.1% (67/88, 95%CI 65.9–84.6%). There was no significant predictive factor of non-PD. (Table 2.3).

**Table 2.3:** Patients received weekly PTX

Factor		Non-PD/N		Univariable analysis	
			Non-PD Rate (95% CI)	Odds ratio (95% CI)	*P*-value
Menopausal status	Premenopausal	24/33	72.7% (54.5%–86.7%)	1	
	Postmenopausal	43/55	78.2% (65.0%–88.2%)	1.349 (0.501–3.633)	0.5532
cT	cT1	3/5	60.0% (14.7%–94.7%)	1	
	cT2	29/36	80.6% (64.0%–91.8%)	2.811 (0.398–19.850)	0.3001
	cT3	6/8	75.0% (34.9%–96.8%)	1.858 (0.176–19.614)	0.6064
	cT4	29/39	74.4% (57.9%–87.0%)	2.008 (0.296–13.635)	0.4757
cN	cN0	7/8	87.5% (47.3%–99.7%)	1	
	cN1	20/27	74.1% (53.7%–88.9%)	0.547 (0.071–4.226)	0.5628
	cN2	13/16	81.3% (54.4%–96.0%)	0.772 (0.084–7.099)	0.8188
	cN3	27/37	73.0% (55.9%–86.2%)	0.524 (0.071–3.848)	0.5252
Visceral metastasis	No	20/27	74.1% (53.7%–88.9%)	1	
	Yes	47/61	77.0% (64.5%–86.8%)	1.198 (0.425–3.383)	0.7324
Histology	Invasive ductal carcinoma	62/80	77.5% (66.8%–86.1%)	1	
	Special types	5/8	62.5% (24.5%–91.5%)	0.465 (0.102–2.115)	0.3221
ER	Negative	43/55	78.2% (65.0%–88.2%)	1	
	Positive	24/33	72.7% (54.5%–86.7%)	0.741 (0.275–1.995)	0.5532
PgR	Negative	46/59	78.0% (65.3%–87.7%)	1	
	Positive	21/29	72.4% (52.8%–87.3%)	0.734 (0.267–2.022)	0.5501
Overall		67/88	76.1% (65.9%—84.6%)		

**Fig. 2.2:**
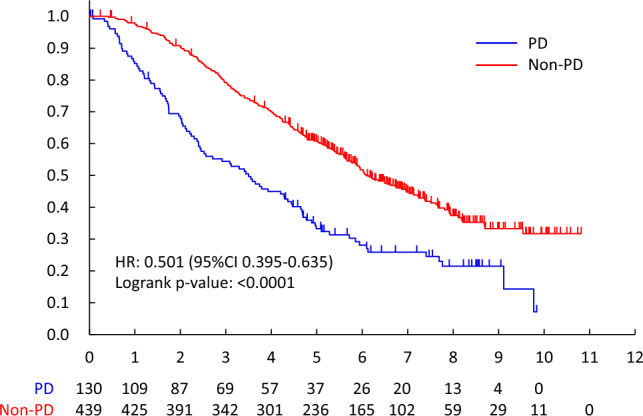
PD vs. non-PD (all patients)

**Fig. 2.3:**
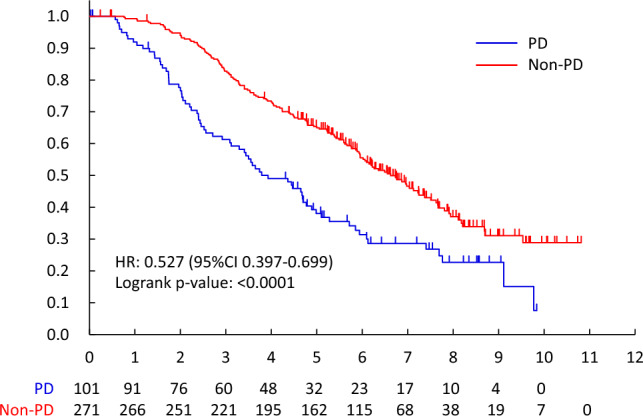
PD vs. non-PD (endocrine therapy)

**Fig. 2.4:**
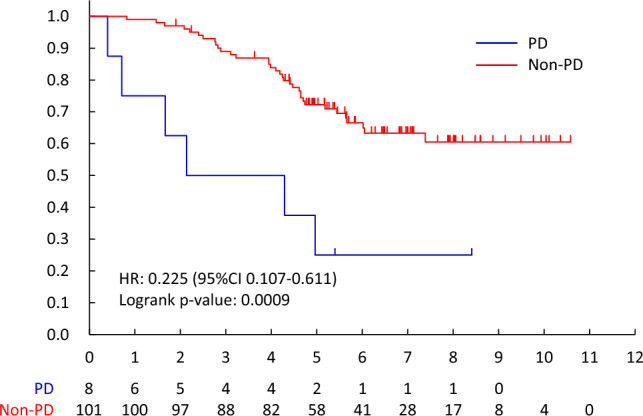
PD vs. non-PD (HPD/HPTX)

**Fig. 2.5:**
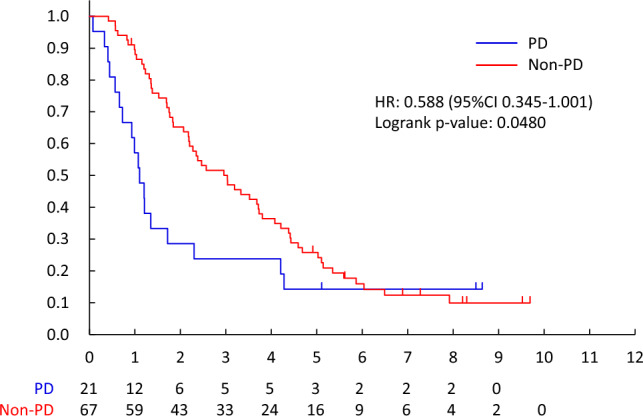
PD- vs. non-PD (PTX)

The non-PD rate for HPD/HPTX was 92.7% (101/109, 95%CI 86.1—96.8%), and that of HPD and HPTX was 92.6% (75/81, 95%CI 84.6–97.2%) and 92.9% (26/28, 95%CI 76.5–99.1%). The predictive factor of non-PD for HPD/HPTX was visceral metastases (OR 15.818, 95%CI 2.547–24.942, p = 0.0030). (Table 2.4) Overall survival was better in patients with non-PD than PD (MST 6.1 years vs. 3.5 years, HR 0.508[95%CI 0.398–0.648], two-sided log-rank p < 0.0001) (Fig. 2). OS between non-PD and PD was significantly different, regardless of drug. (Endocrine therapy; HR 0.532 [95%CI 0.399–0.711], two-sided log-rank p < 0.0001, HPD/HPTX; HR 0.306 [95%CI0.119–0.783], two-sided log-rank p = 0.0088, PTX; HR 0.555 [95%CI0.322–0.956], two-sided log-rank p = 0.0315) (Fig. 2.2, 2.3, 2.4, 2.5).

**Table 2.4:** Patients received HPD/HPTX therapy

Factor		Non-PD/N	Non-PD Rate (95% CI)	Univariable analysis	
				Odds ratio (95% CI)	*P*-value
Menopausal status	Premenopausal	33/35	94.3% (80.8%–99.3%)	1	
	Postmenopausal	68/74	91.9% (83.2%–97.0%)	0.786 (0.170–3.648)	0.7588
cT	cT1	4/4	100.0% (39.8%–100.0%)	1	
	cT2	46/50	92.0% (80.8%–97.8%)	1.147 (0.038–34.742)	0.9370
	cT3	18/19	94.7% (74.0%–99.9%)	1.370 (0.034–54.651)	0.8672
	cT4	33/36	91.7% (77.5%–98.2%)	1.063 (0.034–33.574)	0.9724
cN	cN0	7/7	100.0% (59.0%–100.0%)	1	
	cN1	46/47	97.9% (88.7%–99.9%)	2.067 (0.064–66.654)	0.6821
	cN2	11/15	73.3% (44.9%–92.2%)	0.170 (0.007–4.442)	0.2875
	cN3	37/40	92.5% (79.6%–98.4%)	0.714 (0.028–18.516)	0.8394
Visceral metastasis	No	24/31	77.4% (58.9%–90.4%)	1	
	Yes	77/78	98.7% (93.1%–100.0%)	15.818 (2.547–98.228)	0.0030
Histology	Invasive ductal carcinoma	96/104	92.3% (85.4%–96.6%)	1	
	Special types	5/5	100.0% (47.8%–100.0%)	0.969 (0.038–24.942)	0.9847
ER	Negative	78/84	92.9% (85.1%–97.3%)	1	
	Positive	23/25	92.0% (74.0%–99.0%)	0.778 (0.165–3.682)	0.7520
PgR	Negative	87/93	93.5% (86.5%–97.6%)	1	
	Positive	14/16	87.5% (61.7%–98.4%)	0.431 (0.087–2.131)	0.3019
Overall		101/109	92.7% (86.1%–96.8%)

**Fig. 3:**
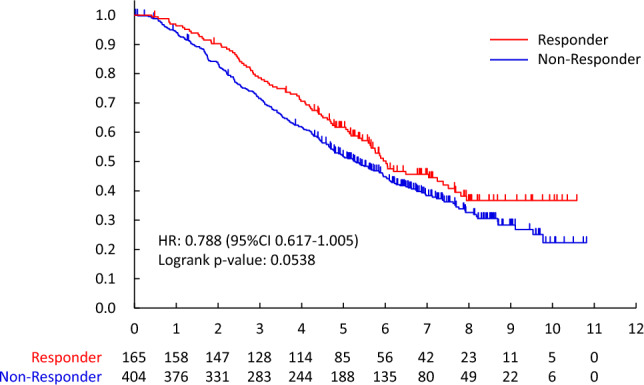
Responder vs. non-Responder (all patients)

According to the RECIST ver. 1.1, the response rate after 3 months systemic therapy for all patients was 29.0% (165/569, 95%CI 25.3–32.9%), and that of endocrine therapy, PTX and HPD/HPTX were 11.6% (43/372, 95%CI 8.5–15.3%), 36.4% (32/88, 95% CI 26.4–47.3%) and 82.6% (90/109, 95%CI 74.1–89.2%). (Table 2.4) The CR in the primary site was only the HPD/HPTX regimen (18/109, 16.5%). The CR in metastatic lesions was 0.5% (2/372), 11.9% (13/109), and 1.1% (1/88) by endocrine, HPD/HPTX, and PTX, respectively. As per the RECIST criteria, OS between responders (CR + PR) and non-responders was not significant (HR 0.788[95%CI 0.617–1.005], two-sided log-rank p = 0.0538). However, there was an important difference between PD and others (CR, PR, and SD) (HR 0.508[95%CI 0.398–0.648]). (Fig. 3).

## Discussion

In this analysis, response predictive factors to PST for non-PD were post-menopausal, PgR positive, and subtype. An ER-/HER2 + tumor was more likely to respond than an ER-/HER2- tumor, while an ER + /HER2- tumor was less likely to respond. The response and predictors of response to PST differed by subtype.

PgR, HER2 expression, and the status of visceral metastasis may have affected the efficacy.

The response rate for first-line treatment with LH-RH agonist and tamoxifen for MBC, including both recurrent and de novo Stage IV BC, is reported to be 38–48%, and the median progression-free survival is 8.7 months [6]. The response rate for first-line treatment with letrozole was 32%, and the median progression-free survival was reported to be 9.4 months [7]. PgR is a target gene of ER, and PgR expression is considered a prognostic predictor of ER-positive BC as an indicator of activation of the ER pathway [8]. More recently, it has also been shown to regulate tumor progression directly, and activation of PgR inhibits breast cancer cell proliferation and is associated with a favorable prognosis [9]. HER2 is a target for molecular targeted drugs. In this trial, endocrine therapy alone was used as first-line treatment in ER-positive HER2-positive BC without life-threatening metastasis. However, this study showed that endocrine therapy was less effective in these subjects. Conversely, patients treated with anti-HER2 drugs showed very high efficacy after 3 months. Therefore, endocrine therapy is not recommended as primary therapy in ER-positive HER2-positive patients. The efficacy of HPD therapy as the primary treatment for HER2-positive metastatic BC was reported in the CLEOPATRA trial [10]. The median PFS was 12.4 months, and the response rate was 80.2%, which is consistent with the results of this study, which reported that HPD is highly effective in ER-positive as well as ER-negative metastatic BC. The efficacy of Taxane (paclitaxel and docetaxel) in HER2-negative metastatic BC is reported to be around a 34% response rate [11]. However, no clinically useful predictors of response to Taxane have been reported. In summary, the results of the present study on the efficacy and predictors of initial drug therapy for de novo Stage IV BC were consistent with those reported previously for recurrent BC as a whole. Therefore, the optimal drug therapy for de novo Stage IV BC should be selected based on the biology and metastatic status of BC, even more so than for recurrent BC. In ER-positive MBC, patients deriving no clinical benefit from first-line endocrine therapy (± CDK4/6 inhibitors) have significantly worse OS than responders. For example, an extensive analysis found median OS ~ 62 months in patients with a first-line objective response versus ~ 41 months in non-responders (p < 0.001) [12]. Primary endocrine resistance is thus a strong negative prognostic factor for survival in this subtype. Similarly, lack of response in HER2 + disease treated first-line with trastuzumab-based regimens (e.g., trastuzumab ± pertuzumab plus chemotherapy) portends inferior survival. Patients achieving an objective response to initial anti-HER2 therapy enjoy markedly longer OS; one study reported median OS 10.2 years in those with complete response vs < 6.3 years in patients with only stable disease [13]. In contrast, those who fail to respond to anti-HER2 therapy face substantially shorter survival. TNBC has the poorest prognosis – median OS is only about 12–18 months with standard first-line chemotherapy [14]. Non-responders to initial therapy (chemotherapy or PARP inhibitors in BRCA-mutant TNBC) have especially short survival, whereas patients who respond can achieve longer OS outcomes [15].

The present study demonstrates that a simple binary classification (non-PD vs. PD) at 3 months is strongly associated with overall survival across all subtypes, suggesting its potential as an early and clinically practical indicator of treatment efficacy. Although simpler than RECIST, this non-PD/PD classification effectively captured prognostic differences and may be helpful in early treatment decision-making.　Since the definition of progression in this study (≥ 10% increase in size) differs from RECIST (≥ 20%), we also compared both classifications. Both identified patients with poor prognosis, but our simplified definition enabled earlier detection of progression, particularly in cases with subtle early enlargement, supporting its pragmatic utility in clinical trials in early response stratification. In addition, incorporating the non-PD concept as an early prognostic indicator may have practical clinical value. Early identification of non-responders could enable timely treatment modification and support adaptive trial designs that dynamically adjust therapy based on early tumor response, thereby improving the translational applicability of this approach.

We uniformly evaluated treatment response at 3 months for all subtypes because the rationale was to provide a consistent timepoint for determining eligibility for primary tumor resection in the main JCOG1017 trial. The optimal timing for assessing maximum drug efficacy may differ among subtypes and treatment types; for instance, endocrine therapy often requires longer exposure than chemotherapy or anti-HER2 therapy. Therefore, while our findings suggest that tumors showing progression within 3 months have significantly worse survival and need early treatment modification, future studies are warranted to define the most appropriate evaluation timing for each subtype. This study was not designed to determine the optimal timing for achieving the maximal response in each subtype, but rather to explore the prognostic and predictive implications of early (3 month) assessment within the context of the JCOG1017 trial. Separate subtype-specific studies will be necessary to address this question in detail.

In this exploratory analysis, we intentionally adopted a more straightforward method than RECIST to facilitate uniform assessment across institutions and to enable timely treatment decision-making. While RECIST and our definition serve different purposes, our results suggest that the more straightforward method still reliably predicted overall survival, underscoring its practical utility for large-scale clinical trials and daily practice.

Finally, it should be noted that this study focused on determining treatment sensitivity and eligibility for primary tumor resection, rather than selecting the optimal initial systemic therapy itself. Further investigations are needed to clarify how these findings can inform initial treatment selection and long-term management strategies in de novo Stage IV breast cancer. We are currently using the same method to evaluate whether local therapy, including metastases, prolongs prognosis in patients with oligometastasis who are sensitive to PST (JCOG2110 trial, jRCTs 031230439, NCT06135714) [16].

There were some limitations in this study. Although new effective drugs have recently been developed to improve prognosis, these drugs were not used in the present study. CDK4/6 inhibitors, standard drugs for ER-positive BC, were also not used. Immune checkpoint inhibitors are used for TN BC, and PARP inhibitors for BRCA mutation-positive BC, but not in this study. Their efficacy or predictive factors are not clear from the results of the present study. On the other hand, in the present study, efficacy was uniformly determined at 3 months for all drugs. This was done to assess the efficacy of the drugs at an early stage and to determine the indication for primary resection. However, it is expected that the maximum efficacy of each drug will not necessarily be 3 months. The present study cannot examine medium- and long-term drug effects. In addition, primary site excision may affect subsequent drug efficacy and prognosis.

## Conclusion

The choice of initial systemic therapy for de novo Stage IV BC is essential to be appropriate for the subtype. Endocrine mono-therapy was not recommended for patients with ER + HER + BC. The prognosis of patients with PD response after 3 months from the start of treatment is poor, and effective systemic therapy needs to be developed for these patients.

## Supplementary Information

Below is the link to the electronic supplementary material.

Supplementary file1 (DOCX 93 KB)

## Data Availability

Individual participant data that underline the results reported in this article will not be shared because the follow-up of the patients is continued until May 2028. After the publication using data as of May, 2029, individual participant data that underlie the results after deidentification will be shared if investigators whose proposed use of the data has been approved by the investigators from Breast Cancer Study Group of JCOG identified for this purpose. Proposals should be directed to tshien@md.okayama-u.ac.jp. The date will be available for achieving aims in the approved proposal.
