# Supplementary material for: Efficacy and prognostic predictors of primary systemic therapy for de novo Stage IV breast cancer. Exploratory analysis of JCOG1017 PRIM-BC
Source: Breast Cancer. 2026 Jan 31;33(2):396–406. doi: 10.1007/s12282-025-01821-4 (PMC12960300; doi:10.1007/s12282-025-01821-4)
Supplement: Supplementary file 1 — Supplementary file1 (DOCX 93 KB) [file 12282_2025_1821_MOESM1_ESM.docx]

***Appendix 1 Participating institutions of JCOG***

NHO Hokkaido Cancer Center, Iwate Medical University, Tohoku University Hospital, Faculty of Medicine, University of Tsukuba, Tochigi Cancer Center, Jichi Medical University, Gunma Prefectural Cancer Center, Saitama Cancer Center, National Cancer Center Hospital East, Chiba Cancer Center, National Cancer Center Hospital, Tokyo Metropolitan Cancer and Infectious Diseases Center Komagome Hospital, National Hospital Organization Tokyo Medical Center, Showa University School of Medicine Cancer Institute Hospital, Toranomon Hospital, St. Luke’s International Hospital, Tokai University School of Medicine, St. Marianna University, Kanagawa Cancer Center, Kitasato University School of Medicine, Yokohama Rosai Hospital, Niigata Cancer Center Hospital, Shizuoka General Hospital, Shizuoka Cancer Center, Aichi Cancer Center Hospital, Nagoya City University Hospital, Nagoya Medical Center, Kinki University School of Medicine, Osaka International Cancer Institute, Osaka National Hospital, Okayama University Hospital, Kure Medical Center Chugoku Cancer Center, NHO Fukuyama Medical Center, Hiroshima University Hospital, Hiroshima City Hospital, Hiroshima City Asa Hospital, NHO Shikoku Cancer Center, National Kyushu Cancer Center, Kitakyushu Municipal Medical Center, Nagasaki Medical Center, Kumamoto University Medical School, Social medical corporation Hakuaikai Sagara Hospital.

Appendix 2


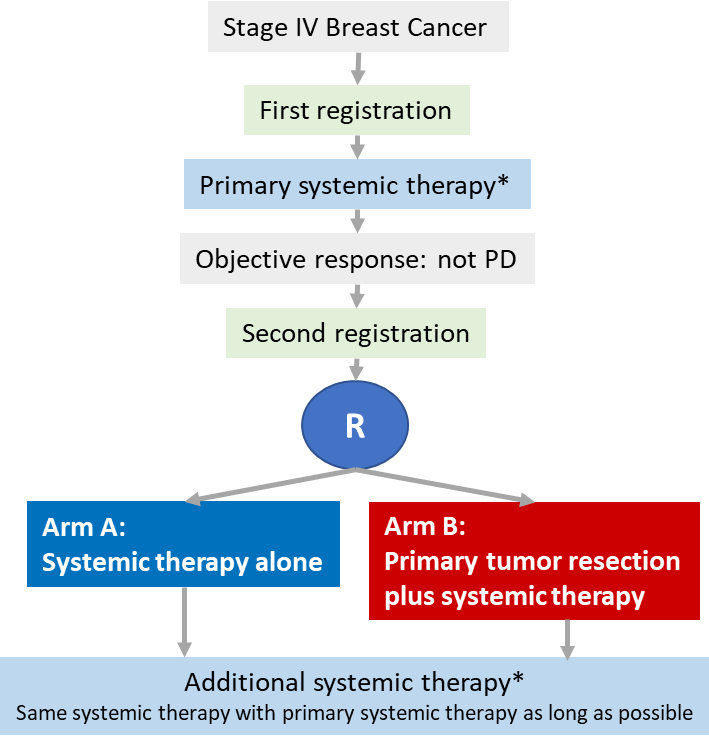


*Primary systemic therapy

**
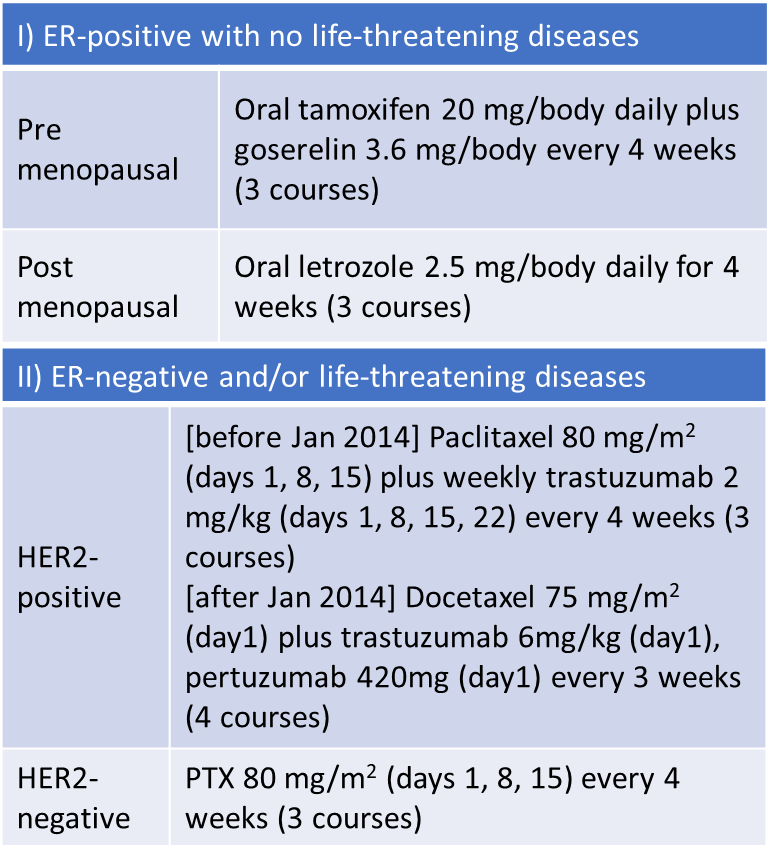
**
